# What a Modern Physician Should Know About microRNAs in the Diagnosis and Treatment of Diabetic Kidney Disease

**DOI:** 10.3390/ijms26146662

**Published:** 2025-07-11

**Authors:** Małgorzata Rodzoń-Norwicz, Patryk Kogut, Magdalena Sowa-Kućma, Agnieszka Gala-Błądzińska

**Affiliations:** 1 Department of Human Physiology, Faculty of Medicine, University of Rzeszów, Al. Tadeusza Rejtana 16C, 35-959 Rzeszów, Poland; pkogut@ur.edu.pl (P.K.); msowa@ur.edu.pl (M.S.-K.); 2 Clinic of Internal Medicine, Nephrology and Endocrinology with Nuclear Medicine Laboratory and Dialysis Center, State Hospital 2 in Rzeszów, Lwowska Street 60, 35-301 Rzeszów, Poland; 3 Department of Nephrology and Endocrinology, Faculty of Medicine, University of Rzeszów, Al. Tadeusza Rejtana 16C, 35-959 Rzeszów, Poland

**Keywords:** diabetic kidney disease, microRNA, biomarkers, therapy, gene regulation

## Abstract

Diabetic kidney disease (DKD) remains the leading cause of end-stage kidney disease (ESKD) globally. Despite advances in our understanding of its pathophysiology, current therapies are often insufficient to stop its progression. In recent years, microRNAs (miRNAs)—small, non-coding RNA molecules involved in post-transcriptional gene regulation—have emerged as critical modulators of key pathogenic mechanisms in DKD, including fibrosis, inflammation, oxidative stress, and apoptosis. Numerous studies have identified specific miRNAs that either exacerbate or mitigate renal injury in DKD. Among them, miR-21, miR-192, miR-155, and miR-34a are associated with disease progression, while miR-126-3p, miR-29, miR-146a, and miR-215 demonstrate protective effects. These molecules are also detectable in plasma, urine, and renal tissue, making them attractive candidates for diagnostic and prognostic biomarkers. Advances in therapeutic technologies such as antagomiRs, mimics, locked nucleic acids, and nanoparticle-based delivery systems have opened new possibilities for targeting miRNAs in DKD. Additionally, conventional drugs, including SGLT2 inhibitors, metformin, and GLP-1 receptor agonists, as well as dietary compounds like polyphenols and sulforaphane, may exert nephroprotective effects by modulating miRNA expression. Recent evidence also highlights the role of gut microbiota in regulating miRNA activity, linking metabolic and immune pathways relevant to DKD progression. Further research is needed to define stage-specific miRNA signatures, improve delivery systems, and develop personalized therapeutic approaches. Modulation of miRNA expression represents a promising strategy to slow DKD progression and improve patient outcomes.

## 1. Introduction

Diabetic kidney disease (DKD) has consistently remained the leading cause of kidney failure worldwide for many years. It develops in approximately 40% of individuals with type 2 diabetes mellitus (T2DM) and about 30% of those with type 1 diabetes mellitus (T1DM) [1,2]. According to the Kidney Disease: Improving Global Outcomes (KDIGO) guidelines, DKD is a clinical diagnosis defined as chronic kidney disease (CKD) in a patient with diabetes mellitus (DM). The current diagnostic criteria for DKD include structural or functional abnormalities of the kidneys that persist for at least three months. Classification of DKD is based on the underlying cause, the estimated glomerular filtration rate (eGFR), and the albuminuria category, expressed as the urinary albumin-to-creatinine ratio (UACR). It should be emphasized that the term diabetic nephropathy (DN) is now reserved for histopathological diagnosis and refers to characteristic diabetic lesions identified in kidney biopsy specimens [3,4].

Hyperglycemia is considered the primary driver of DKD development. Scientific evidence confirms that it induces a range of well-characterized hemodynamic and metabolic alterations [5,6]. In contrast, the impact of the hyperglycemic environment on microRNAs (miRNAs) is currently under intensive investigation and may represent a novel diagnostic tool and therapeutic target in the future [7].

MiRNAs are endogenous, single-stranded, non-coding RNA molecules, 19–23 nucleotides in length, that play a key role in regulating gene expression. The first miRNA was discovered in 1993 in *Caenorhabditis elegans*, where it was shown to regulate larval development. Subsequent studies revealed that miRNAs are widely conserved across eukaryotic species and are fundamental regulators of diverse biological processes [8]. According to miRBase, more than 2600 human miRNAs have been identified to date, collectively regulating approximately 60% of protein-coding genes. MiRNA genes are distributed across all chromosomes except the Y chromosome and are located within transcriptional units (TUs), which can be classified as intronic or exonic [5,9,10].

## 2. MiRNA Biosynthesis

The biosynthesis of miRNA begins in the cell nucleus with the transcription of primary miRNA (pri-miRNA) by RNA polymerase II. The Drosha/DGCR8 complex processes pri-miRNA into precursor miRNA (pre-miRNA), which is then exported to the cytoplasm via exportin-5. In the cytoplasm, the ribonuclease Dicer cleaves pre-miRNA into RNA duplexes, from which one strand (the guide strand) becomes the mature miRNA, while the passenger strand is degraded. The mature miRNA associates with the RNA-induced silencing complex (RISC), which contains Argonaute proteins that guide the miRNA to its target mRNA, leading to its degradation or translational repression [8,9]. 

### MiRNA—Mechanism of Action

miRNAs bind to the 3′ untranslated region (3′UTR) of target mRNAs, resulting in translational repression or transcript degradation, depending on the degree of complementarity. This mechanism represents a key aspect of post-transcriptional gene regulation. A single mRNA can be regulated by multiple miRNAs, underscoring the multilayered control of gene expression [8,9,10].

miRNAs are involved in essential biological processes such as cell differentiation, cell cycle regulation, and apoptosis. Their dysregulation has been implicated in the pathogenesis of cancer, metabolic disorders, and DKD [8,9]. They also play a role in intercellular communication as circulating miRNAs, which are detectable in blood, urine, and cerebrospinal fluid, and are stabilized by protein complexes or transported within exosomes. Due to their stability and specific expression patterns, they are being investigated as potential diagnostic biomarkers [8].

## 3. MiRNAs in DKD

Despite significant advances in understanding the molecular mechanisms underlying DKD, currently available therapies remain only partially effective in halting disease progression. The discovery of the first miRNAs prompted researchers to explore their potential as novel therapeutic targets and biomarkers for monitoring DKD progression. This interest stems from the fact that miRNAs regulate key biological processes such as fibrosis, inflammation, apoptosis, and oxidative stress—all of which are central to DKD pathogenesis [4,9,10].

It is important to note that miRNA expression in DKD is not uniform. Understanding this heterogeneity is crucial, as some miRNAs exert protective effects, while others contribute to kidney injury [4,9,11]. Certain miRNAs are upregulated, amplifying inflammatory responses, whereas others are downregulated, thereby facilitating disease progression [9,11,12].

The recognition that each miRNA plays a unique role in the human body enables a more precise approach to the diagnosis and treatment of DKD. This strategy makes it possible to identify which miRNAs are overactive and require suppression, and which ones could be supported to slow disease progression [9,10,11].

Due to this functional diversity, miRNAs are emerging as valuable biomarker candidates for monitoring patients with DKD and optimizing their treatment. Such an approach would facilitate more personalized patient management, taking into account the individual miRNA expression profile corresponding to specific pathological changes in the kidneys.

## 4. Key miRNAs Involved in DKD Pathogenesis That Promote Kidney Injury

The progression of DKD involves molecular pathways driving fibrosis, oxidative stress, apoptosis, and inflammation. Key miRNAs amplify these processes and contribute to kidney injury and disease progression. The most relevant ones are summarized below.

### 4.1. miR-21

MiR-21 plays a pivotal role in the pathogenesis of DKD by promoting fibrosis and inflammation [13]. Its expression is elevated in the kidneys and plasma of patients with DKD, contributing to accelerated disease progression [12]. By activating the transforming growth factor-beta (TGF-β) pathway, miR-21 enhances collagen synthesis and the production of other extracellular matrix (ECM) components, thereby promoting progressive renal fibrosis [13].

Additionally, miR-21 suppresses three major antifibrotic regulators: tissue inhibitor of metalloproteinases-3 (TIMP3), which controls ECM-degrading enzyme activity; Smad7, which negatively regulates the TGF-β pathway; and phosphatase and tensin homolog (PTEN), a key modulator of cell growth and apoptosis. Inhibition of TIMP3 by miR-21 results in excessive ECM accumulation, Smad7 suppression enhances TGF-β signaling, and PTEN downregulation activates the phosphoinositide 3-kinase/protein kinase B (PI3K/Akt) pathway, promoting fibroblast proliferation and fibrosis [12]. Beyond its profibrotic actions, miR-21 also supports cell proliferation and inhibits apoptosis, further aggravating kidney injury. Given its multifaceted role in DKD pathogenesis, miR-21 is considered one of the most promising therapeutic targets [12,13].

### 4.2. miR-192

Similar to miR-21, miR-192 plays an equally important role in regulating fibrosis and inflammation. In DKD, overexpression of miR-192 stimulates the TGF-β/Smad3 signaling pathway, which increases the production of ECM proteins such as collagen types I and IV. Smad3 is a protein involved in transducing signals initiated by activation of the TGF-β receptor. Once activated, Smad3 becomes phosphorylated and forms a complex with other Smad proteins, most commonly Smad4. This Smad3/Smad4 complex then translocates to the cell nucleus, where it regulates the expression of genes involved in fibrosis, inflammation, and cell proliferation [11]. Moreover, miR-192 decreases the activity of zinc finger E-box-binding homeobox 1 (ZEB1), which suppresses epithelial–mesenchymal transition (EMT); its inhibition promotes excessive ECM accumulation [14]. The activity of miR-192 is particularly complex, as it may support tissue repair in the early stages of DKD, but in advanced stages, its overexpression significantly enhances fibrosis and renal dysfunction [14]. Studies have shown that miR-192 may also induce the expression of proinflammatory cytokines such as monocyte chemoattractant protein-1 (MCP-1) and TNF-α, which are key mediators of inflammation. Furthermore, miR-192 enhances oxidative stress, which further damages renal cells, particularly podocytes and mesangial cells [15]. The regulation of miR-192 is also associated with epigenetic modifications such as DNA methylation and histone acetylation, which influence gene expression. This phenomenon, known as “metabolic memory,” may help explain why certain kidney injuries in the course of DKD continue to progress even after the implementation of intensive glycemic control [11,14].

### 4.3. miR-155

MiR-155 is a well-known regulator of the inflammatory response, activating proinflammatory pathways and promoting immune activation [16]. In DKD, overexpression of miR-155 leads to increased production of proinflammatory cytokines such as IL-6, TNF-α, and MCP-1, fostering chronic inflammation in the kidneys and contributing to injury through oxidative stress, apoptosis, and fibrosis [17]. It also promotes macrophage polarization toward the proinflammatory M1 phenotype, further amplifying renal inflammation [18]. Moreover, miR-155 overexpression activates the nuclear factor kappa-light-chain-enhancer of activated B cells (NF-κB) pathway, contributing to the development of albuminuria [17]. NF-κB is a key signaling pathway in cells, playing a central role in the regulation of inflammatory responses and cell survival. Its activation drives the transcription of genes responsible for the production of cytokines, chemokines, and other inflammatory mediators, which are critical in response to inflammation, cellular stress, and various stimuli [16]. While miR-155 is a major inflammatory regulator, it also contributes to renal fibrosis by modulating the expression of proteins involved in ECM synthesis [18]. In DKD, miR-155 activates the TGF-β/Smad3 pathway, promoting the accumulation of ECM proteins such as collagen and fibronectin, which contributes to the progression of fibrosis and glomerulosclerosis [19]. miR-155 also inhibits the expression of antifibrotic factors such as PTEN, which under physiological conditions limits fibroblast proliferation and ECM production [18]. By blocking PTEN, miR-155 facilitates excessive fibroblast activation, thereby exacerbating renal fibrosis [19].

### 4.4. miR-34a

MiR-34a contributes to the pathogenesis of DKD by regulating inflammation, oxidative stress, and apoptosis. Its expression is significantly elevated in the kidneys of patients with DM, suggesting a critical role in the pathophysiology of DKD. Overexpression of miR-34a induces podocyte apoptosis by promoting the generation of reactive oxygen species (ROS) and inhibiting the expression of anti-apoptotic proteins such as B-cell lymphoma 2 (Bcl-2) [20]. In addition, miR-34a exerts its effects by inhibiting the expression of silent information regulator 1 (SIRT1), a protein that protects cells from oxidative stress and apoptosis [21]. Inhibition of SIRT1 leads to excessive activation of the p53 protein, which promotes cell apoptosis and amplifies inflammatory processes [20]. In DKD, the upregulation of miR-34a and the consequent downregulation of SIRT1 accelerate ECM accumulation, contributing to fibrosis [21]. Furthermore, the SIRT1 reduction increases cellular susceptibility to hyperglycemia-induced oxidative stress, further enhancing podocyte injury [20,21].

### 4.5. miR-217

In DKD, miR-217 promotes renal fibrosis primarily by inhibiting the expression of SIRT1, a protein that protects against oxidative stress, apoptosis, and fibrosis. This leads to the activation of proinflammatory signaling pathways such as TGF-β/Smad3, which drive the synthesis and deposition of ECM proteins, including collagen. It also contributes to excessive fibroblast proliferation, increased ECM production, and progressive renal fibrosis, a key process in DKD progression. MiR-217 appears to play a particularly important role in advanced stages of the disease, where its expression correlates with intensified fibrosis and podocyte apoptosis [22].

### 4.6. miR-423-5p

The expression of miR-423-5p is increased in DKD, particularly in more advanced stages of the disease, and contributes to disease progression by regulating several key signaling pathways involved in renal fibrosis and inflammation. It modulates the TGF-β/Smad3 pathway, leading to Smad3 activation and increased transcription of genes encoding ECM proteins such as collagen types I and IV [23]. MiR-423-5p may also affect the activity of NF-κB, resulting in increased expression of proinflammatory cytokines including TNF-α and IL-6, thereby intensifying renal inflammation, promoting cellular injury, and accelerating DKD progression [24]. It can further inhibit the PI3K/Akt pathway, which plays a key role in cell growth and proliferation. Suppression of this pathway leads to increased apoptosis of podocytes and mesangial cells [24]. In addition, miR-423-5p directly regulates the expression of genes encoding ECM proteins such as collagen type I and fibronectin, promoting ECM accumulation in glomeruli and renal tubules, which results in sclerosis and gradual loss of kidney function [23]. miR-423-5p also increases the expression of NADPH oxidase 4 (NOX4), leading to excessive ROS production and enhanced oxidative stress in podocytes, contributing to their injury [25]. A study focused on the role of long non-coding RNA (lncRNA) XIST in DKD demonstrated that XIST functions as a “molecular sponge” for miR-423-5p, binding and reducing its availability. As a result, miR-423-5p—normally involved in suppressing the expression of proliferation-promoting genes—becomes less effective, leading to increased levels of various proteins such as high mobility group AT-hook 2 (HMGA2), which contribute to renal injury. HMGA2 is a regulatory protein that binds DNA and alters its three-dimensional structure. It interacts with DNA via “AT-hook” motifs, thereby modulating the accessibility of other transcription factors and influencing chromatin organization necessary for gene regulation [23].

### 4.7. mir-214

MiR-214 plays a key role in the pathogenesis of DKD by promoting pathological processes such as fibrosis, oxidative stress, inflammation, apoptosis, and excessive proliferation of mesangial cells [26]. Its increased expression in the kidneys of patients with DKD contributes to enhanced tissue damage and accelerates disease progression [27]. In promoting fibrosis, miR-214 activates the TGF-β/Smad3 signaling pathway, leading to excessive extracellular matrix (ECM) deposition [26]. Studies suggest that miR-214 directly targets genes involved in this pathway, thereby influencing the progression of renal fibrosis [28]. Elevated miR-214 levels have been associated with increased oxidative damage in DKD, although some studies suggest that miR-214 may also exert protective effects under specific conditions [27,28]. The imbalance in redox signaling may contribute to the activation of pro-fibrotic pathways such as TGF-β/Smad3 [26]. In addition, miR-214 may regulate the expression of proinflammatory cytokines, contributing to increased activation of inflammatory mediators such as NF-κB, which controls the release of cytokines including IL-6, TNF-α, and IL-1β [26]. Studies have also shown that miR-214 overexpression increases caspase activity, key enzymes responsible for apoptosis [27]. Excessive apoptosis in the kidneys leads to structural damage of glomeruli and renal tubules and, together with mesangial cell hyperproliferation, contributes to the progressive loss of kidney filtration function [26,27,28].

### 4.8. miR-199a

MiR-199a plays a significant role in the pathogenesis of DKD, affecting fibrosis, oxidative stress, and apoptosis. Some studies have shown that miR-199a contributes to renal fibrosis by modulating the TGF-β/Smad3 signaling pathway and suppressing antifibrotic proteins such as caveolin-1 [29]. Moreover, evidence suggests that TGF-β can activate the p53 protein, which, in turn, upregulates miR-199a-3p. This leads to the suppression of SOCS7 and activation of the STAT3 pathway—a mechanism strongly associated with renal fibrogenesis [30]. The p53 protein, often referred to as the “guardian of the genome,” plays a central role in regulating the cell cycle and cellular stress responses. Sustained activation of p53 and miR-199a may result in caspase activation and increased apoptosis, thereby exacerbating kidney injury in DKD [30].

### 4.9. miR-193a

MiR-193a plays an important role in the pathogenesis of DKD, particularly through its impact on podocyte function [31]. In DKD, the expression of miR-193a is typically elevated, leading to podocyte injury through enhanced apoptosis and structural and functional disruption [32]. Specifically, miR-193a promotes the loss of essential podocyte proteins such as nephrin and podocin, which impairs the glomerular filtration barrier, resulting in proteinuria and progressive decline in glomerular filtration [31]. Studies have shown that miR-193a significantly affects the regulation of transcription factors such as Wilms’ Tumor 1 (WT1), which is essential for maintaining podocyte homeostasis [32]. Increased expression of miR-193a in DKD suppresses WT1, leading to podocyte apoptosis and the exacerbation of degenerative processes in the kidney [31]. Podocyte loss is one of the major mechanisms contributing to kidney failure in DKD, making miR-193a a key regulator in disease progression [32]. Moreover, miR-193a not only affects podocytes but also impacts other renal cells by promoting fibrosis and inflammation. By modulating pathways related to proinflammatory cytokines, it contributes to glomerular structural changes and tubular degeneration. Consequently, elevated miR-193a activity drives DKD progression [31,32].

**Figure 1:**
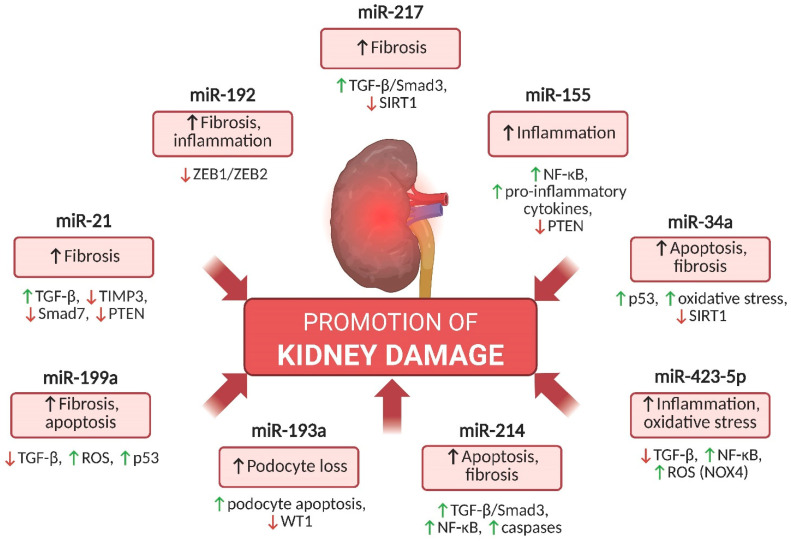
Pathogenic miRNAs in DKD. Schematic representation of selected miRNAs (e.g., miR-21, miR-192, miR-155) that contribute to DKD progression by promoting renal fibrosis, inflammation, podocyte loss, apoptosis, and oxidative stress. Each miRNA regulates specific signaling pathways, including TGF-β/Smad3, NF-κB, p53, and others, which collectively contribute to the deterioration of kidney function. Abbreviations: miRNA—microRNA; DKD—diabetic kidney disease; TGF-β—transforming growth factor beta; NF-κB—nuclear factor kappa B; PTEN—phosphatase and tensin homolog; MCP-1—monocyte chemoattractant protein-1; TNF-α—tumor necrosis factor alpha; ROS—reactive oxygen species; WT1—Wilms tumor 1; NOX4—NADPH oxidase 4; SIRT1—sirtuin 1. ↑ indicates upregulation or activation; ↓ indicates downregulation or inhibition.

In Figure 1, the major miRNAs promoting kidney injury in DKD are summarized, along with a brief description of their mechanisms of action.

## 5. Key miRNAs with Protective Potential in DKD

Some miRNAs exert nephroprotective effects by regulating fibrosis, oxidative stress, apoptosis, and inflammation. They may slow DKD progression by modulating inflammation, preserving endothelial function, and limiting extracellular matrix buildup. Key protective miRNAs are summarized below.

### 5.1. miR-126-3p

In DKD, the downregulation of miR-126-3p contributes to microvascular dysfunction, increased endothelial permeability, and progression of tissue damage [33,34]. These effects are partially mediated through its regulation of the HIF-1α/VEGF signaling axis, which normally maintains vascular integrity and limits pathological angiogenesis [35]. Additionally, miR-126-3p modulates endothelial cell survival, proliferation, and inflammation by influencing pathways such as AKT/HK2 and NF-κB. Its deficiency has been linked to enhanced production of reactive oxygen species (ROS), increased levels of proinflammatory cytokines, including IL-6 and TNF-α, and sustained activation of inflammatory responses in the diabetic kidney [33,34].

### 5.2. miR-29

MiR-29 acts as a potent antifibrotic regulator by controlling the expression of genes involved in the production of collagen and other ECM components [36]. In particular, it inhibits the expression of genes encoding collagen types I, III, and IV (COL1A1, COL1A2, COL3A1, COL4A1), which are major constituents of ECM. In DKD, reduced miR-29 expression leads to excessive ECM deposition, contributing to the progression of renal fibrosis [37]. MiR-29 also influences the balance between cell proliferation and death by modulating signaling pathways related to apoptosis. Under oxidative or inflammatory stress, conditions characteristic of DKD, miR-29 helps limit excessive cell apoptosis, thereby preventing further kidney damage [36]. Additionally, miR-29 inhibits pathways associated with chronic inflammation and oxidative stress, both of which play key roles in DKD progression [37]. By regulating the expression of genes linked to proinflammatory mediators and ROS production, miR-29 helps mitigate oxidative and inflammatory damage in the kidney.

### 5.3. miR-451

MiR-451 serves as an important regulatory factor in DKD, influencing inflammatory mechanisms and oxidative stress, which play a central role in disease progression [38,39]. Its expression is reduced in the kidneys of patients with DKD and correlates with increased inflammation and podocyte degradation [38]. One of the main mechanisms of miR-451 activity is the regulation of the NF-κB pathway, which governs inflammatory responses. In the context of DKD, reduced expression of miR-451 leads to excessive production of proinflammatory cytokines such as IL-6 and TNF-α, contributing to chronic renal inflammation and ongoing tissue injury [38]. MiR-451 also plays a role in controlling oxidative stress by limiting the accumulation of ROS, which damage endothelial cells and podocytes. Its downregulation in DKD increases cellular susceptibility to oxidative injury, further promoting disease progression [39].

### 5.4. miR-30a

In DKD, the expression of miR-30a is typically reduced, contributing to the activation of the TGF-β/Smad3 pathway, which promotes ECM deposition and leads to renal fibrosis [40]. One of the key mechanisms of miR-30a activity is the regulation of apoptosis. miR-30a inhibits the B-cell lymphoma 2/Bcl-2-associated X protein (Bcl-2/Bax) pathway, limiting caspase activation and thereby suppressing apoptosis, particularly in podocytes [41]. In DKD, reduced miR-30a expression results in enhanced apoptosis, contributing to podocyte degeneration and glomerular damage, ultimately impairing the kidney’s filtration function [40]. MiR-30a is also an important regulator of oxidative stress. Its downregulation in DKD contributes to increased ROS production via activation of NADPH oxidase 4 (NOX4) [41]. In addition, miR-30a plays a role in suppressing inflammation, and its decreased expression during DKD leads to activation of the NF-κB pathway, a central regulator of inflammatory responses [40].

### 5.5. miR-146a

MiR-146a plays a key role in regulating inflammation and oxidative stress in DKD. As a modulator of inflammatory responses, it primarily acts through the NF-κB pathway, which controls the expression of proinflammatory cytokines such as IL-6 and TNF-α. Under hyperglycemic conditions, miR-146a suppresses excessive inflammatory responses; thus, its decreased expression in DKD is associated with intensified inflammation and disease progression [42]. In addition, miR-146a influences other inflammatory pathways, including tumor necrosis factor receptor-associated factor 6 (TRAF6) and interleukin-1 receptor-associated kinase 1 (IRAK1). Under conditions of elevated oxidative stress, miR-146a regulates these pathways, reducing ROS production and thereby limiting cellular damage. Ultimately, the downregulation of miR-146a in DKD may contribute to chronic inflammation, exacerbating renal fibrosis and functional decline [42].

### 5.6. miR-215

MiR-215 contributes to the pathogenesis of DKD by influencing signaling pathways related to cell proliferation, apoptosis, and renal fibrosis. Its protective effects stem from the suppression of connective tissue growth factor (CTGF) expression, which reduces fibrotic processes that are central to DKD progression [43]. Through modulation of the PI3K/Akt pathway, miR-215 also regulates cell proliferation by inhibiting excessive pathway activation [44]. In DKD, reduced levels of miR-215 are associated with increased activity of proinflammatory and proapoptotic pathways, accelerating renal injury [43]. MiR-215 further acts by regulating the TGF-β/Smad2 signaling pathway, one of the major drivers of fibrosis in DKD [44]. Increased activity of this pathway, resulting from low miR-215 expression, contributes to excessive ECM deposition and structural disruption of the glomeruli [43]. Additionally, miR-215 is involved in the regulation of apoptosis. Its reduced expression in the kidneys during DKD leads to increased activation of proapoptotic pathways such as Bcl-2/Bax, promoting excessive cell death—particularly in podocytes and tubular epithelial cells. This contributes to their degeneration and further impairment of renal filtration function [44].

**Figure 2:**
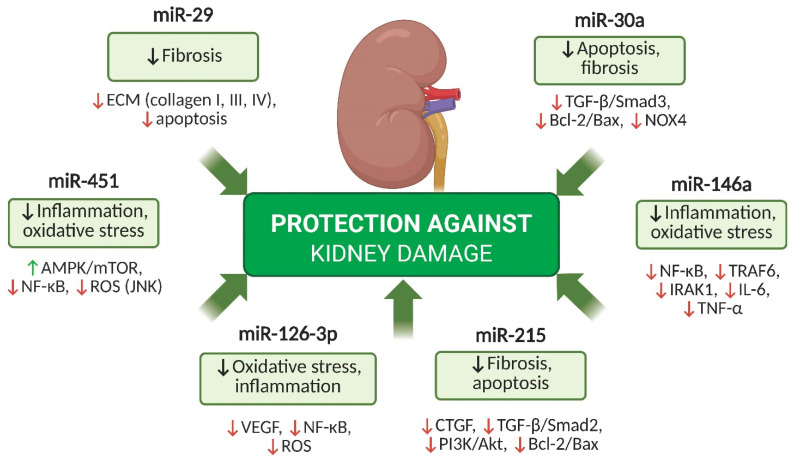
Protective miRNAs in DKD. Overview of selected miRNAs (e.g., miR-29, miR-126-3p, miR-146a, miR-215) that exert renoprotective effects in DKD. These miRNAs reduce inflammation, oxidative stress, fibrosis, and apoptosis through the regulation of signaling pathways such as TGF-β/Smad, NF-κB, VEGF, and PI3K/Akt, thereby preserving kidney structure and function. Abbreviations: miRNA—microRNA; DKD—diabetic kidney disease; TGF-β—transforming growth factor beta; NF-κB—nuclear factor kappa B; ROS—reactive oxygen species; VEGF—vascular endothelial growth factor; NOX4—NADPH oxidase 4; AMPK—AMP-activated protein kinase; mTOR—mechanistic target of rapamycin; JNK—c-Jun N-terminal kinase; TRAF6—TNF receptor-associated factor 6; IRAK1—interleukin-1 receptor-associated kinase 1; IL-6—interleukin-6; TNF-α—tumor necrosis factor alpha; CTGF—connective tissue growth factor; PI3K—phosphoinositide 3-kinase; Akt—protein kinase B; Bcl-2—B-cell lymphoma 2; Bax—Bcl-2-associated X protein. ↑ indicates upregulation or activation; ↓ indicates downregulation or inhibition.

In Figure 2, the major miRNAs with protective potential in DKD and their mechanisms of action are presented.

## 6. MiRNAs as Potential Biomarkers in DKD

MiRNAs have gained significant attention as potential biomarkers in DKD due to their stability in body fluids, specific expression patterns, and their ability to regulate key pathological processes such as inflammation and fibrosis. Their use in the diagnosis and monitoring of DKD may be based on detecting miRNAs in various biological samples, including plasma, urine, and kidney biopsy material. Although blood is the most accessible source of circulating miRNAs, their diagnostic specificity for DKD is limited. For instance, miR-21 is involved in multiple non-renal pathologies, including cardiovascular, hepatic, and oncological diseases [35]. Therefore, urinary or biopsy-derived miRNAs may more accurately reflect kidney-specific injury and pathophysiological processes. While various studies have shown that individual or combined miRNA expression patterns can differentiate DKD patients from controls with moderate to high sensitivity and specificity, these results are often context-dependent and require further validation. For instance, miR-192, miR-21, and miR-29a have demonstrated diagnostic utility with area under the curve (AUC) values ranging from 0.78 to 0.92 in selected cohorts. However, variability in patient populations, biological sample types (e.g., blood vs urine), and analytical methods limits the generalizability of these findings. Therefore, miRNAs may serve best as components of multiparametric diagnostic panels rather than as standalone biomarkers [35,45,46].

Importantly, miRNAs do not act in isolation; rather, they function as part of dynamic regulatory networks. The simultaneous upregulation of some miRNAs (e.g., miR-21, miR-192) and downregulation of others (e.g., miR-29, miR-126-3p) reflects the complexity of molecular responses in DKD, including fibrosis, inflammation, and endothelial dysfunction. These diverse expression patterns indicate that a combinatory approach—analyzing multiple miRNAs together—may provide a more accurate disease signature than evaluating individual markers alone [25,35,45,46,47,48].

The altered expression of miRNAs in DKD is not random but driven by multiple upstream signals related to the diabetic milieu. Chronic hyperglycemia, oxidative stress, activation of the RAAS, and proinflammatory cytokines such as TNF-α, IL-6, and TGF-β are known to modulate the transcription of miRNA genes. For example, TGF-β signaling has been shown to upregulate profibrotic miRNAs such as miR-21 and miR-192 via SMAD-mediated transcriptional activation. Conversely, oxidative stress and hypoxia may downregulate protective miRNAs like miR-29 or miR-126-3p. Epigenetic mechanisms such as promoter methylation and histone modifications also contribute to miRNA dysregulation in DKD. These upstream stimuli integrate to alter miRNA expression profiles, ultimately amplifying inflammation, fibrosis, and tubular apoptosis within the diabetic kidney microenvironment [46,48,49].

While several studies support that damage-associated miRNAs (e.g., miR-21, miR-192) are upregulated during DKD progression and protective miRNAs (e.g., miR-29, miR-126-3p) are downregulated, these patterns are not entirely consistent across all patient populations and disease stages. Some protective miRNAs may be partially restored following therapeutic interventions or during disease stabilization, but this has not been universally observed. Factors such as tissue specificity, timing of sample collection, and coexisting conditions (e.g., cardiovascular disease, hypertension) can influence miRNA profiles. Therefore, although certain expression trends are well-documented, miRNAs should be interpreted as context-dependent markers rather than absolute indicators of progression or remission [45,46,47,48,49].

**Figure 3:**
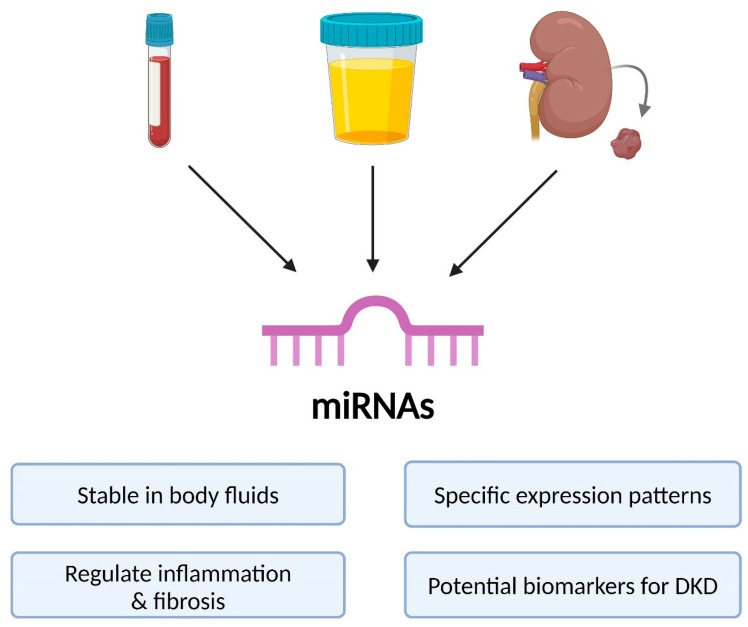
Sources and functions of miRNAs in DKD. Diagram illustrating the biological sources of circulating miRNAs, which can be detected in blood, urine, and renal biopsy specimens. Owing to their stability in body fluids and specific expression profiles, miRNAs have potential as diagnostic and prognostic biomarkers in DKD. They regulate key pathological processes such as inflammation and fibrosis, making them relevant tools for disease monitoring and risk stratification. Abbreviations: miRNA—microRNA; DKD—diabetic kidney disease.

MiRNAs circulate in body fluids in two major forms: as protein-bound molecules (e.g., bound to Argonaute proteins) or enclosed within exosomes, which protect them from degradation. Exosomes are extracellular vesicles involved in miRNA transport between cells, ensuring their stability and playing a role in intercellular communication [50,51]. These materials—blood, urine, and kidney biopsy specimens—are also depicted in Figure 3, which illustrates the main biological sources used for miRNA detection in DKD. For their detection in DKD diagnostics, methods such as quantitative real-time PCR (qPCR), next-generation sequencing (NGS), and microRNA microarrays (miRNA arrays) are used [52].

Moreover, specific miRNAs may appear at different stages of DKD progression, offering insights not only into diagnosis but also into disease staging. For instance, miR-192 and miR-126-3p have been shown to change early in DKD, even before the onset of albuminuria, making them promising early biomarkers [45,47]. In contrast, miR-21, miR-155, and miR-29 are typically dysregulated in later stages and reflect fibrotic and inflammatory responses [46,48,49].

Preclinical studies indicate that miR-192 is a marker of early kidney injury, and its increased expression correlates with disease progression [45]. MiR-21 is closely associated with fibrosis and inflammation, and its overexpression reflects advanced stages of DKD [49,53]. MiR-29 has antifibrotic properties, and its downregulation is linked to progressive ECM accumulation in the kidney [46]. MiR-126-3p is being investigated as a biomarker of vascular injury, with reduced plasma levels in DKD patients correlating with endothelial dysfunction and the development of vascular complications [47]. MiR-146a exhibits anti-inflammatory effects, and its decreased expression is associated with intensified renal inflammation [48]. MiR-215 and miR-217 are involved in the regulation of cell proliferation and fibrosis—alterations in their levels may indicate disease advancement and progression of DKD [54,55,56].

Although many miRNAs discussed in this review—such as miR-21, miR-192, miR-29, and miR-146a—are dysregulated in both T1DM and T2DM, their expression levels and pathophysiological roles may differ between these groups. In T1DM, miR-146a appears to play a more prominent role in modulating immune and inflammatory responses, particularly through the NF-κB pathway [57]. In contrast, in T2DM, metabolic and profibrotic miRNAs such as miR-21 and miR-192 are more consistently associated with the development and progression of DKD [45,58]. These differences likely reflect the distinct pathophysiological mechanisms driving each diabetes type, including autoimmune versus metabolic-inflammatory profiles [35]. Recognizing these distinctions may help tailor future miRNA-based diagnostics and therapeutics more precisely to diabetes subtype.

A summary of miRNA functions and their clinical utility in DKD diagnostics is presented in Table 1. It highlights seven miRNAs with the strongest and most consistent diagnostic evidence, while other miRNAs mentioned in the text are still under preliminary investigation or lack sufficient clinical validation.

While most studies included in this review were conducted in patients with already established DKD, they do not allow for a definitive conclusion as to whether dysregulated miRNA expression is a cause or a consequence of the disease. Some animal studies suggest that certain miRNAs, such as miR-21 or miR-192, may act upstream by promoting inflammation and fibrosis, thus contributing to the initiation of kidney damage [46,49,58]. On the other hand, many changes in miRNA profiles likely represent a downstream effect of chronic metabolic stress or tissue remodeling. Further longitudinal studies are needed to clarify these relationships and determine which miRNAs may serve as early drivers versus passive markers of DKD.

Although individual miRNAs, such as miR-21, miR-192, and miR-29, exert distinct roles in inflammation or fibrosis, they do not operate in isolation but are part of interrelated regulatory networks. These networks involve both synergistic and antagonistic interactions, and some miRNAs may indirectly regulate others via shared signaling pathways. For instance, miR-21 and miR-192 are both induced by TGF-β signaling, which also downregulates antifibrotic miR-29 through SMAD-dependent mechanisms [46,49]. Similarly, inflammatory pathways such as NF-κB may repress protective miRNAs like miR-146a while activating pathogenic ones. This suggests a hierarchical structure in which upstream signals modulate key miRNAs, which, in turn, influence the expression or stability of others through feedback loops or common targets. Understanding this regulatory crosstalk may help identify nodal miRNAs that act as master regulators of fibrosis, inflammation, and apoptosis in DKD and could therefore serve as prioritized therapeutic targets.

## 7. Preclinical Models and Functional Validation of miRNAs in DKD

Several animal models have been employed to functionally validate the role of individual miRNAs in DKD. These models demonstrate that miRNAs are not merely biomarkers but active contributors to DKD pathogenesis, especially in the regulation of inflammation, fibrosis, and apoptosis. Podocyte-specific knockout models have been used to study the effects of miR-192 deficiency, showing reduced extracellular matrix accumulation and protection against early glomerular injury in diabetic settings [46]. AntagomiR-based suppression of miR-21 in streptozotocin-induced diabetic mice led to reduced renal fibrosis and improved kidney function, supporting its profibrotic role in vivo [49,58]. Similarly, locked nucleic acid (LNA)-modified antisense oligonucleotides targeting miR-192 have been shown to decrease the expression of profibrotic genes such as collagen 1α1 and TGF-β1 in diabetic mouse models [46]. In addition, the delivery of miR-126-3p via exosome-based nanocarriers has demonstrated renoprotective effects, including attenuation of tubular apoptosis and reduction of inflammatory cytokine production in murine DKD models [47]. These findings confirm that targeted modulation of miRNA expression in vivo is not only feasible but can significantly alter disease progression. Therefore, miRNA-based therapies represent a promising translational avenue for DKD intervention and merit further preclinical and clinical investigation.

## 8. MiRNAs as Therapeutic Targets in DKD: Perspectives and Challenges

MiRNAs represent promising therapeutic targets in DKD, and their modulation may help reduce fibrosis, inflammation, and oxidative stress. This can be achieved using antagomiRs, which inhibit the activity of overexpressed miRNAs, or antisense oligonucleotides (ASOs), which bind to miRNAs to block their function or modulate their regulation [59]. Locked nucleic acids (LNAs) stabilize ASO molecules, increasing their affinity for target miRNAs and enhancing therapeutic effects [59]. miRNA sponges sequester miRNAs, preventing their interaction with mRNAs, thereby enabling control over multiple miRNAs simultaneously. Conversely, miRNA mimics restore the function of downregulated miRNAs, potentially counteracting fibrosis and inflammation [59,60]. Advanced technologies, such as exosomes and nanoparticles, enable precise delivery of miRNAs to target tissues, protecting them from degradation and increasing therapeutic efficacy [61,62]. Various strategies for miRNA modulation in DKD are currently being tested in preclinical studies. MiR-21, whose overexpression promotes fibrosis, is one of the most extensively studied miRNAs in DKD. Inhibition of its activity using antagomiR-21 reduced ECM accumulation and improved kidney function [58]. Similarly, miR-192 activates the TGF-β pathway and promotes fibrosis—its inhibition using LNA decreased collagen expression (COL1A1, COL3A1) [63]. MiR-215 regulates inflammation, oxidative stress, and fibrosis. MiR-215 mimics reduced collagen and proinflammatory cytokine expression, while nanoparticle delivery enhanced therapeutic efficacy [64]. MiR-126-3p influences endothelial function and angiogenesis, and its mimics improved renal function and reduced albuminuria, partly through VEGF modulation. Exosomes containing miR-126-3p effectively reduced inflammation and apoptosis in renal endothelial cells [65,66]. MiR-29 is a key regulator of fibrosis, and its reduced expression promotes ECM accumulation. MiR-29 mimics inhibited TGF-β activation and reduced fibrosis, while ASO and nanoparticle strategies aimed at increasing miR-29 expression have shown efficacy in DKD models [67,68]. Although research on miRNAs in DKD remains at the preclinical stage, their therapeutic potential is significant—particularly in reducing fibrosis, protecting the endothelium, and limiting oxidative stress.

## 9. The Impact of Commonly Available Drugs and Dietary Compounds on miRNA Modulation in DKD

An increasing number of studies suggest that drugs and dietary compounds may modulate miRNA expression, thereby influencing key processes in the pathogenesis of DKD, including inflammation, oxidative stress, and fibrosis. Although the underlying mechanisms are not yet fully understood, miRNA regulation may contribute to the nephroprotective effects of many therapeutic strategies.

### 9.1. SGLT2 Inhibitors

SGLT2 inhibitors exert pleiotropic protective effects in DKD, including reduction of oxidative stress, suppression of inflammation and fibrosis, improvement of glomerular hemodynamics, and reduction of albuminuria [69]. A key mechanism underlying their action involves the modulation of the NF-κB and TGF-β signaling pathways, which play a fundamental role in regulating miRNAs associated with DKD progression [70]. It has been suggested that SGLT2 inhibitors may modulate the expression of miR-21, miR-29, and miR-146a, which could help explain their nephroprotective effects [69,70].

### 9.2. GLP-1 Receptor Agonists

GLP-1 receptor agonists demonstrate potential nephroprotective effects, including reduction of oxidative stress, improvement of endothelial function, and inhibition of renal fibrosis [71]. This mechanism may result from their influence on the NF-κB pathway, suggesting regulation of inflammation-related miRNAs [72]. It has been proposed that they may downregulate miR-21, thereby limiting fibrosis, and increase miR-126 levels, promoting vascular stability and improving endothelial function [71,72].

### 9.3. Statins

In addition to their lipid-lowering properties, statins exhibit anti-inflammatory and antifibrotic effects, suggesting a potential influence on miRNA expression [57,73]. By inhibiting the NF-κB pathway, they may reduce miR-21 levels, thereby attenuating activation of proinflammatory and profibrotic signaling and restoring the function of peroxisome proliferator-activated receptor alpha (PPARα), which is essential for lipid metabolism and mitochondrial homeostasis [73].

### 9.4. Metformin

Beyond its classical metabolic effects, metformin may modulate the expression of miRNAs involved in inflammation and fibrosis. It has been suggested that metformin affects miR-155-5p by inhibiting the Toll-like receptor 4/nuclear factor kappa-light-chain-enhancer of activated B cells (TLR4/NF-κB) axis, potentially reducing the production of proinflammatory cytokines and slowing DKD progression [74]. Reports also indicate a potential impact on other miRNAs, including miR-21 and miR-192, which may be relevant in the context of oxidative stress and apoptosis [75].

### 9.5. Polyphenols

Polyphenols may modulate miRNA expression, potentially contributing to their anti-inflammatory, antioxidant, and antifibrotic effects [76,77]. Resveratrol and quercetin may inhibit miR-21, which could reduce inflammation and fibrosis [78]. Epigallocatechin-3-gallate (EGCG) and quercetin upregulate miR-29, a key regulator of ECM remodeling and renal protection against fibrosis [78]. Additionally, resveratrol and curcumin may modulate miR-146a and miR-155, both of which are involved in the regulation of inflammatory responses and oxidative stress, thereby potentially limiting chronic inflammation in DKD [76].

### 9.6. Sulforaphane

Sulforaphane, a compound naturally found in cruciferous vegetables, exhibits nephroprotective properties by inhibiting the TGF-β/Smad pathway and downregulating miR-21, thereby reducing fibrosis and inflammation [79]. At the same time, it activates the Nrf2 pathway and increases the expression of miR-29, contributing to renal structural protection and decreased ECM accumulation [79].

### 9.7. The Role of Gut Microbiota in miRNA Modulation in DKD

The gut microbiota is a complex ecosystem of microorganisms that helps maintain systemic homeostasis. In T2DM, dysbiosis increases the production of trimethylamine N-oxide (TMAO) and reduces levels of short-chain fatty acids (SCFAs), promoting inflammation, oxidative stress, and DKD progression [80,81,82,83]. Evidence suggests that gut microbiota influences miRNA expression involved in proinflammatory and profibrotic pathways [84]. Probiotics (e.g., Lactobacillus, Bifidobacterium) may reduce inflammation and uremic toxins such as p-cresyl sulfate (PCS) and indoxyl sulfate (IS) [85], while prebiotics (e.g., inulin) promote anti-inflammatory bacteria and may indirectly affect miRNA expression [81,86]. Changes in microbiota have been associated with elevated levels of miR-122-5p, which regulates lipid metabolism and inflammatory responses, as well as miR-122 and miR-33, which are involved in glucose metabolism and DKD progression [84,87]. Modulating gut microbiota and miRNA expression may offer new therapeutic strategies.

## 10. Summary

DKD is a leading cause of end-stage kidney disease (ESKD), with progression linked to inflammation, fibrosis, and oxidative stress [4,27]. miRNAs have emerged as key regulators of these processes, positioning them as promising biomarkers and therapeutic targets [5,27]. miR-21 and miR-29 are central to fibrosis, while miR-146a regulates inflammation [5,9,19]. AntagomiRs and mimics offer therapeutic potential by modulating miRNA activity, possibly slowing DKD progression [6,19,27]. Drugs and bioactive compounds may also influence miRNA expression, expanding therapeutic options [5,9,11]. The gut microbiota may affect miRNA regulation, linking it to DKD pathogenesis and therapy [27,80]. Future research should identify stage-specific miRNAs and refine delivery systems using exosomes and nanotechnologies [27,61]. Real-time miRNA sequencing could enhance therapy monitoring [5,19]. Personalized strategies based on miRNA profiles and genetic variability may improve treatment efficacy and patient outcomes [11,27].

## Figures and Tables

**Table 1:** miRNAs with biomarker potential in DKD. Summary of seven miRNAs with the strongest and most consistent clinical evidence supporting their diagnostic or prognostic utility in DKD. Each miRNA is associated with specific pathological mechanisms, including fibrosis, inflammation, apoptosis, or endothelial dysfunction, and may serve as a marker of disease onset, progression, or stage-specific complications. Abbreviations: DKD—diabetic kidney disease; miRNA—microRNA.

miRNA	Biomarker Function	Clinical Application in Diagnostic DKD
miR-192	Regulator of fibrosis	High expression in early stages; marker for early kidney damage; potential therapeutic target
miR-21	Regulator of fibrosis and inflammation	Indicator of disease progression and fibrosis; potential therapeutic target
miR-29	Anti-fibrotic activity	Decreases with disease progression; potential early-stage marker
miR-126-3p	Endothelial function and vascular health marker	Associated with vascular complications and endothelial health; potential indicator of vascular damage in DKD
miR-146a	Anti-inflammatory function	Reduced levels in advanced DKD; potential therapeutic target
miR-215	Fibrosis regulator	Promising early-stage biomarker; indicator of disease progression
miR-217	Cell apoptosis regulator	Associated with various DKD stages; potential marker for cellular health and disease progression
